# Biomechanical Analysis of an Elite Para Standing Cross-Country Skier Using Lower Limb Prostheses: A Case Study

**DOI:** 10.3390/s26010149

**Published:** 2025-12-25

**Authors:** Cristina De Vito, Cristian Pasluosta, Patrick Ofner, Leonie Hirsch, Natalie Mrachacz-Kersting, Uwe Kersting, Thomas Stieglitz, Walter Rapp, Laura Gastaldi

**Affiliations:** 1 Department of Mechanical and Aerospace Engineering, Politecnico di Torino, 10129 Turin, Italy; laura.gastaldi@polito.it; 2 Laboratory for Biomedical Microtechnology, Department of Microsystems Engineering (IMTEK), Albert-Ludwigs-Universität Freiburg, 79110 Freiburg, Germany; cristian.pasluosta@imtek.uni-freiburg.de (C.P.); patrick@ofner.science (P.O.); thomas.stieglitz@imtek.uni-freiburg.de (T.S.); 3 BrainLinks-BrainTools Center, Albert-Ludwigs-Universität Freiburg, 79110 Freiburg, Germany; natalie.mrachacz-kersting@sport.uni-freiburg.de; 4 Institute of Sport and Sport Science, Albert-Ludwigs-Universität Freiburg, 79110 Freiburg, Germany; leo.a.hirsch@gmail.com; 5 Institute of Biomechanics and Orthopaedics, German Sport University Cologne, 50933 Cologne, Germany; u.kersting@dshs-koeln.de; 6 Olympic Training Center, 79117 Freiburg, Germany; walter.rapp@osp-freiburg.de

**Keywords:** para cross-country skiing, prosthetics, IMUs, diagonal style, cycle spatiotemporal parameters, joint angles

## Abstract

Para cross-country (XC) skiing has become a prominent sport since its debut at the Örnsköldsvik Winter Olympic Games in 1976. Nevertheless, the lack of studies focusing on standing para XC skiing highlights the need to provide a comprehensive description of this sport, investigating how different prosthetic devices may influence the athletic outcome. In this exploratory case study, the biomechanics of an elite standing para-athlete, with a right-sided transfemoral amputation, was investigated. Tests were performed during diagonal XC skiing on a treadmill, at different speeds and inclinations. Specifically, two different prosthetic feet were compared: the athlete used an Ottobock Genium X3 prosthetic knee with either the Ottobock Taleo or the Ottobock Evanto prosthetic foot. Inertial Measurement Units (IMUs) were employed to estimate joint angles and detect pole hits and lifts. Additionally, data were collected using embedded sensors in the knee prosthesis. Diagonal stride spatiotemporal parameters were further calculated. Results revealed that the Evanto foot significantly increased swing phase duration and hip range of motion, while generating higher knee torque, ankle torque, and axial loading compared to the Taleo foot. This research represents the first application of the employed testing methodology to para standing XC skiing, and it therefore provides a framework for future studies on this discipline.

## 1. Introduction

Para cross-country (XC) skiing was introduced at the inaugural Winter Paralympic Games in Örnsköldsvik in 1976 [1]. Over the past decades, para XC skiing has evolved remarkably, and the number of participating athletes and nations has steadily increased, reflecting both growing accessibility and interest [2]. Notably, the popularity of para cross-country skiing continues to grow both as a recreational activity and as a competitive sport [2]. During XC skiing competitions, athletes usually face tracks with uphill, flat, and downhill segments [3]. Athletes may compete by alternating different skiing styles, according to their individual preferences, terrain condition, the required speed and other internal and external factors [4,5,6,7]. These techniques include the classic ones (diagonal stride, double poling with leg kick, double poling and herringbone technique) and the newer freestyle ones (diagonal skate or Gear I, V1-skating or Gear II and V2-skating or Gear III) [8]. Particularly, the diagonal stride technique, which is used both on flat terrain and uphill, involves arms and legs moving in opposition to each other, with the arm push-off coupled with the contralateral leg push-off [9]. Two main phases for the arm movement can be identified: the poling phase (pole ground contact) and the recovery phase (arm swing) [9,10]. Similarly, leg action can be divided into a contact phase and a swing phase [9,10]. The leg contact phase can be further divided into a gliding phase and the thrust phase or push-off phase [9,10].

Paralympic XC skiers are divided, under the rules of the International Paralympic Committee (IPC), into three categories: sitting, standing and visual impairments [3]. To ensure fairness and equality within each category, athletes are further grouped into classes which indicate the functional impact disability has on skiing performance [3]. Physically impaired standing skiers belong to eight different classes (locomotor winter—LW): classes LW2–4 group athletes with lower limb impairments, classes LW5–8 group athletes with upper limb impairments and classes LW9 combines upper and lower limb disabilities [3]. Specifically, athletes with only one lower limb affected by an impairment (i.e., an above knee amputation) belong to class LW2, whereas athletes with both lower limbs affected belong to class LW3 (i.e., muscle weakness in both legs) [3]. Class LW4 groups skiers with only one lower limb affected but with a less severe impact than class LW2 (i.e., below knee amputation or loss of muscle control in one leg) [3]. Additionally, a skier with a unilateral lower limb deficiency may choose to ski standing, with or without a prosthesis [11].

Para-sports provide significant benefits by promoting physical health, psychological well-being and social integration. However, practicing para XC skiing presents significant challenges for athletes with lower limb amputations. In able-bodied skiers, efficient XC skiing relies on precise balance control, rapid adaptations to uneven terrain, and effective force transmission through the lower limbs [11]. In contrast, lower limb amputees’ biomechanics may be affected by increased joint stiffness [12], asymmetrical weight-bearing, a reduced base of support and the decreased ability of generating high propulsive forces [13]. These challenges primarily affect lower-limb amputees in their daily activities [14,15,16]; consequently, they may become even more pronounced in a sport like XC skiing, which requires a high degree of coordination to maintain balance, efficiency and propulsion. Therefore, prosthetic technology plays a crucial role in addressing these biomechanical challenges. Microprocessor-controlled knees (MPKs) modulate joint kinematics through real-time adjustment of knee flexion-extension, employing integrated sensors and adaptive control algorithms to ensure stance phase stability and controlled knee motion during highly dynamic activities [17,18]. The mechanical properties of the prosthetic foot directly affect the lower limb kinematic chain. Energy Storage and Return (ESR) feet, characterized by carbon fibre composite structures, store elastic strain energy during the movement’s loading phase and release it during push-off, potentially increasing propulsive forces compared to rigid Solid Ankle Cushion Heel (SACH) feet [19]. However, most commercially available ESR feet function as single-axis mechanisms constrained to medio-lateral axis rotation, limiting their compliance along the antero-posterior and inferior-superior axes [20]. Conversely, multiaxial feet enable triaxial motion that accommodates varying terrain conditions [20]. This enhanced adaptability may be particularly advantageous in XC skiing, where athletes experience continuously varying slopes and snow surface conditions.

Recent studies have investigated performance characteristics and technique selection in physically impaired standing skiers. The effect of class, sex, and final rank on time distribution across terrains during para XC skiing races has been reviewed in [21]. In [22], race time analyses revealed that para cross-country skiers exhibited significantly larger performance variations compared to able-bodied athletes, suggesting disability-related factors contribute to greater competitive variability. A framework for in-field analyses of performance and sub-technique selection in standing para cross-country skiers was proposed in [4], providing insights into technique distribution and performance metrics during competitions. Additionally, musculoskeletal simulations of XC skiing were performed in [23] as a complementary tool for the classification of athletes with physical impairments. While these studies have investigated performance metrics, race dynamics, technique selection and classification at the class level, in-depth biomechanical analyses focusing on individual standing para-athletes is still missing. Moreover, previous biomechanical research on prosthetic limbs for use in sport has primarily focused on running [12,24] and long jumping [24]. In cycling, prosthetic adaptations were shown to affect pedalling work and range of motion [25], while in alpine skiing, multi-axis prosthetic knees demonstrated movement kinematics similar to intact lower limbs [26]. However, comprehensive biomechanical analyses of XC skiing with prosthetic devices remain absent from the literature. To enhance athletes’ performance, refine training methodologies, and guide equipment design, it is crucial to first understand the biomechanics of para standing XC skiing with different leg prostheses. Characterizing how different prosthetic devices affect movement biomechanics may reveal where potential improvement exists and may establish the specific performance metrics against which design choices and technical adaptations can be evaluated. 

Given the absence of prior biomechanical analyses comparing prosthetic combinations in elite XC para skiing, this study was designed as an exploratory investigation. The aim of this work was to conduct a biomechanical analysis of an elite XC para standing skier with above knee amputation, when performing diagonal stride on a treadmill, using different foot prostheses. Therefore, two prosthetic combinations were compared: the Genium X3 knee prosthesis with the Taleo foot (X3T) and with the Evanto foot (X3E). Secondly, biomechanical adaptations across different speed-inclination conditions were analyzed. Specifically, lower limb joint angles were estimated for both the prosthetic limb and the sound limb, using inertial measurements units (IMUs). Poling cycles were identified using an IMU positioned on the right wrist, which detected characteristic acceleration patterns during pole plant and release. The evaluated cycle parameters included both the poling and recovery phases of the right arm, as well as the stance and swing phases of the prosthetic foot, determined using a force sensor embedded in the distal shank pylon of the knee prosthesis. Finally, kinetics and kinematics data collected through the sensors integrated in the prosthetic knee were analysed.

## 2. Materials and Methods

### 2.1. Participant 

This investigation was conducted as a single-participant case study, a design choice commonly employed in elite para-sport research due to the limited athlete population and the high heterogeneity of impairments. A male elite cross-country para-skier (27 years old, 75 kg, 182 cm), volunteered to participate in the study. We verified the athlete’s testing eligibility according to the following criteria: (i) age ≥ 18 years; (ii) presenting a unilateral above-knee amputation; (iii) regular use of a prosthesis for cross-country skiing; and (iv) sufficient cognitive capacity to understand the study procedures and comply with all requirements. Exclusion criteria consisted of any acute injury or medical condition that could compromise performance or the safety of the experimental protocol.

The athlete was a unilateral right transfemoral amputee classified as LW2, with 15 years of competitive experience in cross-country skiing. Prior to the measurements, the participant was informed about the aims and procedures of the study and signed informed consent. The measurements were performed in accordance with the ethical principles of the Declaration of Helsinki.

### 2.2. Prosthetic Devices

The Genium X3 knee prosthesis (Ottobock Healthcare, Duderstadt, Germany) was tested in combination with two different prosthetic feet: the Taleo prosthetic foot (Ottobock Healthcare, Duderstadt, Germany) and the Evanto prosthetic foot (Ottobock Healthcare, Duderstadt, Germany). Genium X3 is a monocentric microprocessor controlled prosthetic knee, which offers customizable modes, for recreational or sport activities (e.g., XC skiing) [27,28]. These customizable modes can be pre-configured and activated by the user via motion patterns or a mobile application [27]. For the present study, the participant used the configuration he usually adopts when skiing and he was most familiar with. Two controller parameters were adjusted accordingly: “Basic Flexion Damping” was set at 70 and “Increase Damping” was set at 42 (manufacturer’s settings). The Taleo foot is an ESR prosthetic foot [19,29]. It provides limited passive compliance primary in the sagittal plane through the deflection of its carbon composite structure. In contrast, the Evanto foot is an ESR multi-axial foot with up to 20° of sagittal plane motion, ±10° of mediolateral flexibility, and ±4° of torsional motion [30,31]. The multi-axial design of the Evanto foot allows for more mobility in multiple directions, which could be beneficial for a greater range of foot rotation and adduction/abduction.

### 2.3. Measurement Instrumentation

#### 2.3.1. Inertial Measurements Units

For the kinematic measurements, the Trigno Avanti™ System (Delsys^®^, Natick, MA, USA) was employed. This system includes wireless sensors, each equipped with a built-in inertial measurement unit comprising a triaxial accelerometer, gyroscope, and magnetometer [32]. 

Only predefined acquisition modes can be selected using the Trigno Discover software (Delsys^®^, Natick, MA, USA) [32], with each sensor operating in only one mode at a time and providing a specific subset of kinematic variables. To obtain complete kinematic data for each body segment, two adjacent sensors were employed in different modes (Table 1):

- The “IMU Mode” providing triaxial linear acceleration from the accelerometer and triaxial angular velocity from the gyroscope;
- The “Orientation Mode” providing 3D orientation calculated onboard through sensor fusion of the accelerometer, gyroscope and magnetometer data [32].

**Figure 1:**
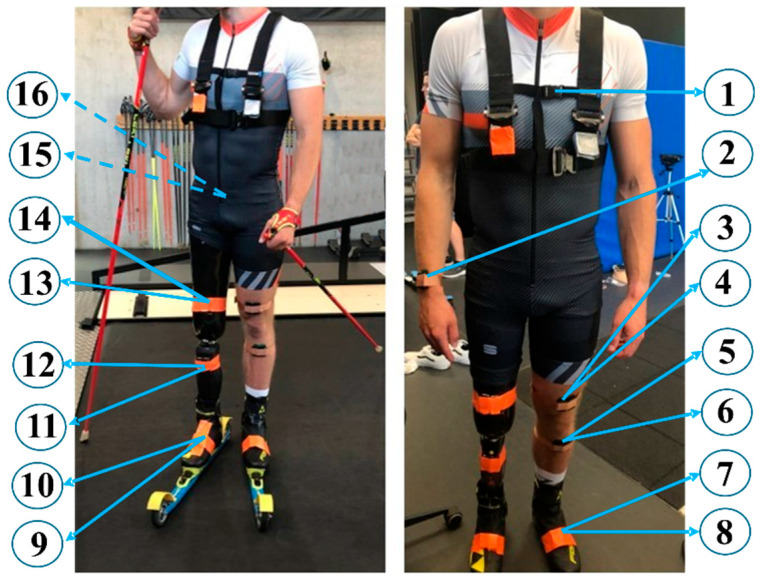
Subject wearing IMUs and safety harness with (**left**) and without skis (**right**). Dashed arrows indicate where the IMUs were placed on the back of the subject, while continuous arrows indicate the location of the sensors on the front of the subject. Circled numbers indicate the reference number of each sensor (Table 1).

The “IMU Mode” was set with the default sampling rate of 370 Hz, while in the “Orientation Mode” data was sampled at 222 Hz. This frequency was selected as the highest available in this mode, since 370 Hz was not supported [32]. To capture linear acceleration, angular velocity and orientation, two sensors were placed next to each other on the following segments: sacrum, sound thigh, sound shank, sound foot, socket case, prosthesis and prosthetic foot (Figure 1). On each segment, one sensor was set to operate in “IMU Mode”, while the adjacent unit operated in “Orientation Mode”. Moreover, it was necessary to collect inertial data from the trunk and the right wrist to estimate the sensor-to-segment alignment and skiing cycle segmentation, respectively. For this purpose, a single sensor operating in “IMU Mode” at 370 Hz was placed on each of these segments.

#### 2.3.2. Embedded Sensors in the Knee Prosthesis

The Genium X3 system features an IMU located at the knee joint, on the shank segment. The sensing system consists of a biaxial accelerometer and a single-axis gyroscope. The accelerometer measures linear acceleration components along the prosthetic shank longitudinal axis and the perpendicular antero-posterior axis contained within the sagittal plane. The gyroscope detects angular velocity around the medio-lateral axis, oriented orthogonally to the sagittal plane of motion. Additionally, an embedded axial magneto resistive encoder at the joint measured the knee angle over time, from which the knee flexion-extension angular velocity was derived by calculating the first derivative over time. An additional strain gauge-based sensor on the distal end of the prosthesis’ hydraulic system was used to evaluate knee loading, while human body axial loading was recorded by a strain gauge-based sensor in the distal shank pylon [33]. Synchronization between the external IMUs and the sensors embedded in the prosthesis was achieved using event-based synchronization. The following data were collected by the internal prosthesis controller at the default sampling rate of 100 Hz and transmitted via Bluetooth to a computer:

- Flexion-extension knee torque;
- Flexion-extension ankle torque;
- Knee flexion-extension angular velocity;
- Knee flexion-extension angle;
- Shank angular velocity;
- Shank angle: prosthesis orientation in the sagittal plane, calculated through proprietary sensor fusion algorithms (Ottobock) applied to the embedded IMU signals.
- Axial load: human body axial load in the direction of the prosthesis measured at the distal shank pylon.

### 2.4. Testing Protocol 

All the measurements were conducted in a controlled setting in the laboratory of the Olympic Training Centre in Freiburg, Germany. 

**Figure 2:**
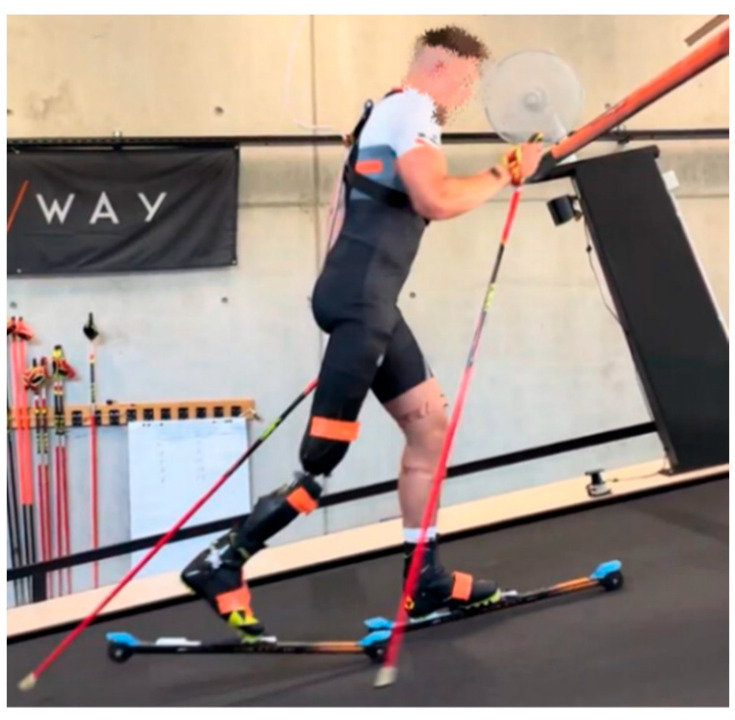
Participant performing diagonal XC skiing at 6 km/h, 22% inclination.

The participant performed diagonal stride roller-skiing on a treadmill (S-Mill, motek-forcelink, Coulembourg, The Netherlands) during a single testing session (Figure 2). The athlete wore standard athletic training clothing. All IMUs sensors were securely attached directly to the skin using hypoallergenic adhesive tape to minimize movement artifacts (Figure 1). A total of 23 trials were collected at different speeds (ranging from 8 km/h to 10 km/h) and inclinations (ranging from 8% inclination to 22% inclination) that were ecologically valid and could be performed accurately by the athlete. An additional familiarisation phase with the system was performed at the beginning of the recording. Please note that we analysed only a subset of trials (see below). 

Before beginning the experimental protocol, the participant performed a standardized 10-min warm-up. Prior to the testing of each prosthetic combination, a dataset for sensor-to-segment alignment was collected, following the procedure described in [27]. To assess the local reference frame, the participant was asked to perform three squats, three trunk rotations to the right, three trunk rotations to the left, three left hip adduction/abduction repetitions, three right hip adduction/abduction repetitions and to stand in a neutral position for at least 10 s.

The participant’s own Genium X3 prosthesis fitted with the Taleo foot was tested first, using the XC skiing controller configuration that he regularly uses during training. The participant was instructed to perform at least twenty poling cycles per trial and to kick on the ground with the prosthetic limb, holding onto the frontal support of the treadmill, at the beginning and end of each trial. The resulting impact peaks were used as synchronization events between the prosthetic sensors and external IMUs to temporally align data from the two measurement systems. Between each trial, the treadmill was not stopped, but speeds and inclinations were changed while the subject was holding to the treadmill support in front of him.

After completing all trials with the X3T configuration, a 20-min rest period was provided. This rest period was considered sufficient to prevent fatigue and no signals of physical or mental exhaustion were observed or reported by the participant. Afterwards, the prosthetic foot was changed to the Evanto foot by a certified orthopedic technician following standardized clinical alignment procedures. This was done off the treadmill and off the skis and included a 20-min familiarization period. The same testing protocol was then repeated with the X3E configuration. 

### 2.5. Data Analysis

Data were analysed using MATLAB software (R2024b, The MathWorks Inc., Natick, MA, USA).Three matching trials were selected for each prosthetic foot, with identical speed and inclination combinations that were representative of competitive racing scenarios familiar to the athlete, thereby ensuring ecological validity. Specifically, the selected conditions reflected three distinct terrain demands encountered in cross-country skiing: a low inclination of 8% at 10 km/h, an intermediate inclination of 17% at 7 km/h, and a steep inclination of 22% at 6 km/h.

#### 2.5.1. Local Reference Frame Assessment 

The positioning of the sensors was assessed according to the procedure described in [34]. This method was implemented to identify the rotation matrix that allows the transformation from the sensor reference frame to the corresponding anatomical reference frame for each body segment. The anatomical coordinate system was defined with the Y-axis vertical pointing upwards in the longitudinal direction, the X-axis pointing forwards in the anteroposterior direction and the Z-axis pointing to the right in the mediolateral direction. The following assumptions were applied to the functional movements performed as described in Section 2.4: (i) squat movements occurred around the medio-lateral axis; (ii) trunk rotations were performed along the vertical axis; (iii) the trunk segment was vertical during upright posture (i.e., no flexion or lateral bending). These assumptions were used to calculate the rotation necessary to align each sensor frame with its corresponding anatomical reference frame of the segment [34]. In more detail, a principal component analysis (PCA) was conducted on the angular velocity data of each sensor to identify its principal rotation axes. The rotation matrix required to align these axes with the anatomical ones was then computed and applied to each sensor [34]. The sensors placed on the right wrist and on the feet were excluded from this procedure [34]. Instead, gravity alignment was applied to the sensors on the right wrist and on the feet, as described in [35]. 

#### 2.5.2. Cycle Events Detection

Firstly, external IMU acceleration raw data were downsampled to 100 Hz. Secondly, external IMUs were synchronized with the embedded sensors in the knee prostheses using vertical acceleration signals from both sensor types. Peaks in the signals (caused by the athlete’s foot hitting the floor) marked the start of acquisition. 

**Figure 3:**
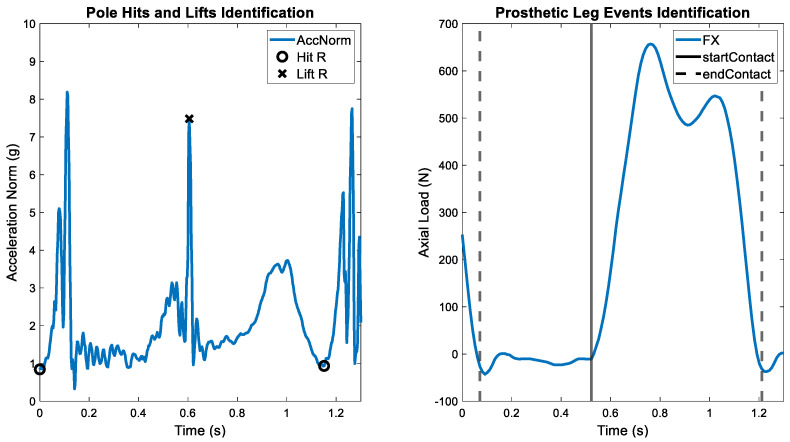
Example of an identification of poling cycle events for the Genium X3+Taleo combination at 8% and 10 km/h. On the right wrist’s acceleration norm (AccNorm) the pole hit of the right (R) arm and the pole lift of the right arm were marked (**left**). On the axial load signal (FX), the start contact of the prosthetic foot (startContact) and the lift of the foot (endContact) were identified (**right**).

Four event types were identified for each poling cycle in each trial (Figure 3): pole hit, pole lift, the start of foot contact phase and the end of foot contact phase (corresponding to the end of the foot swing phase and its start, respectively). The norm of the acceleration signal from the right wrist’s IMU was used to identify the pole hit and pole lift events as follows. When the pole comes to the ground, a peak in the acceleration norm is observed [36]. A second peak appears in the signal when the wrist is raised to lift the pole [36]. Accordingly, the detection of the hit peaks was conducted using a threshold-based method [36]. Similarly, the lift peak was identified by locating the maximum acceleration norm within a trial-specific frame window, starting from the previously identified hit indices [36]. 

The Genium axial load (FX) signal was used to determine the start and end of foot contact. Positive values in the FX signal indicated weight-bearing during the contact phase [33], while negative values appeared during foot lifting due to inertial forces. Thus, negative peaks in the force signal marked the end of the contact phase and the lifting of the prosthetic foot. First, to identify the end of contact events, the signal was inverted and negative peaks were detected using a minimum peak height of 65 N and a minimum peak distance of 100 samples. The start of each contact phase was determined by identifying subsequent peaks relative to the detected end of contact events. Only peaks exceeding a trial-specific threshold (between 5 and 50 N) were considered and this search was conducted within a trial-specific window of 5–20 samples following the end of contact event. Finally, to refine this detection, the start of the contact phase was defined as the sample closest to a force of zero within a window delimited by the initially identified start of contact and the previous end of contact one.

#### 2.5.3. Estimation of Poling Cycle Spatiotemporal Parameters

The duration of each poling cycle was determined as the time interval between two successive pole hit events. For the right arm, the duration of the poling phase was calculated as the time difference between one pole hit and the subsequent pole lift. The swing phase duration was determined as the time difference between the total cycle time and the poling phase time. For the prosthetic foot, the contact phase duration was calculated as the time difference between the start of contact frame and the subsequent end of contact. The duration of the swing phase was calculated as the time difference between the total cycle time and the contact phase duration. Each phase was also expressed as a percentage of the overall poling cycle (%TC). Finally, the cycle length was indirectly calculated as the product of the cycle time and the treadmill velocity. 

#### 2.5.4. Joint Angle Evaluation

The orientation of each sensor was obtained using the strapdown integration method and the drift correction proposed in [37,38]. Under the assumption that each IMU was rigidly attached to the underlying segment, the relative motion between sensors was evaluated. The linear acceleration and angular velocity data collected from each sensor in IMU mode were used for this analysis. The method described in [39] was implemented to determine the relative orientation between the quaternions of each sensor at each time point. Specifically, the sensors on the pelvis and on the thigh were used to estimate the hip joint angle, while the sensors on the thigh and the shank were used to evaluate the knee angle. Lastly, the sensors on the shank and on the foot were used to determine the ankle angles. The obtained relative orientation between two sensors was later converted into Euler angles using the ZYX convention [40]. Maximum flexion-extension angle, minimum flexion-extension angle and flexion-extension range of motion (ROM) were evaluated for each poling cycle of the six trials analyzed.

#### 2.5.5. Kinetics and Kinematics Data of Leg Prosthesis

The data acquired with the sensors embedded in the Genium X3 prosthesis (see Section 2.3.2) were analysed and their maximum value, minimum value, and range (defined as the absolute difference between maximum and minimum) were computed for each poling cycle identified in the six trials analysed.

#### 2.5.6. Statistical Analysis 

Statistical analysis was performed using MATLAB software (The MathWorks Inc.). Fifteen consecutive diagonal stride poling cycles, starting from the third cycle, were analysed in each trial. For each cycle, the following parameters were computed: spatiotemporal metrics (phases of foot contact, foot swing, arm poling, and arm recovery), as well as maximum, minimum, range for joint angles and data from the embedded prosthesis sensors (see Section 2.3.2). Since the data were not normally distributed, tested using Shapiro–Wilk test, the differences between the two prosthetic combinations (Genium X3+Taleo vs. Genium X3+Evanto) were determined with the two-sided Wilcoxon Signed Rank Test and corrected for multiple comparisons using the False Discovery Rate (FDR) method [41]. A *p*-value less than 0.05 was considered statistically significant. Finally, mean flexion-extension angles and their standard deviation were estimated by averaging the fifteen poling cycles considered.

## 3. Results

### 3.1. Spatiotemporal Parameters

**Figure 4:**
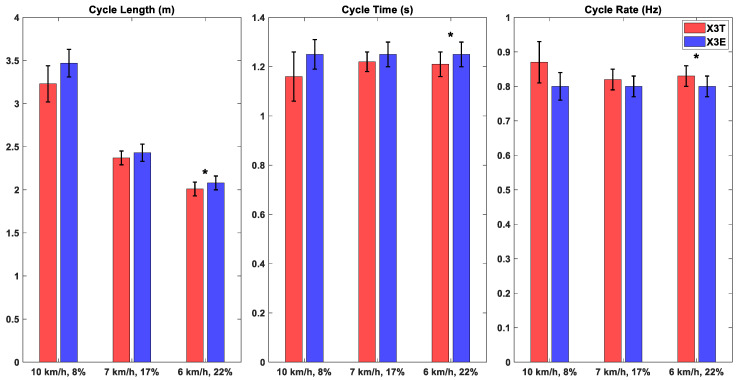
Mean and standard deviation (Std) of cycle spatiotemporal parameters. Statistical significance between Genium X3+Taleo (X3T) and Genium X3+Evanto (X3E) is indicated through asterisks: * *p*-value < 0.05, ** *p*-value < 0.01, *** *p*-value < 0.001.

The percentage of the poling cycle spent in the foot contact phase was significantly higher for the X3T than for the X3E combination at all three inclinations (Table 2). The average duration of the poling cycles ranged between 1.16 and 1.25 s for all the trials (Figure 4). The mean cycle rate ranged between 0.80 Hz and 0.87 Hz across all trials. The average cycle length ranged between 2.01 and 3.47 m. At all three inclinations and speed combinations, Genium X3T exhibited the shortest cycle duration. As expected, the cycle time increased as the cycle rate decreased (Figure 4). 

### 3.2. Joint Angles 

**Figure 5:**
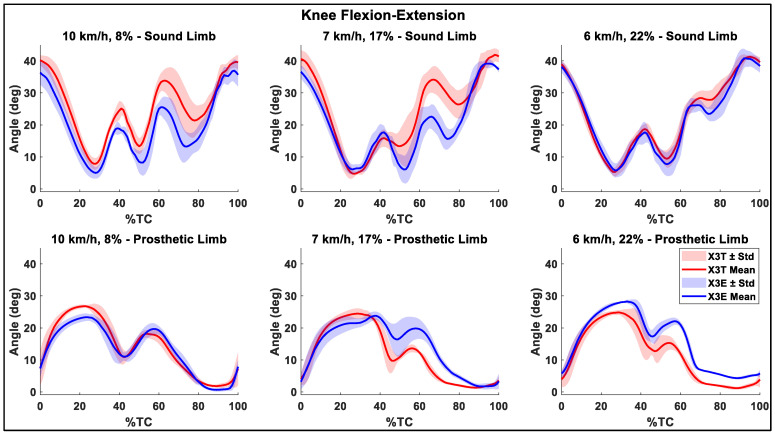
Mean knee flexion-extension angle and standard deviation (Std) band. The first row shows the angles for the sound leg side, while the second row presents the results for the prosthetic side. The three columns, each consisting of two subplots, correspond to the three inclination-velocity combinations tested. In each subplot the results for both the Genium X3+Taleo (X3T) and the Genium X3+Evanto (X3E) are shown. The cycle duration was normalized to 100%.

**Figure 6:**
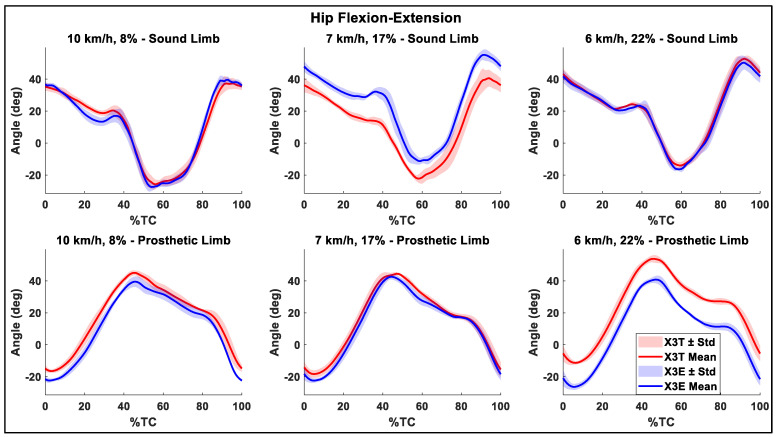
Mean hip flexion-extension angle and standard deviation (Std) band. The first row shows the angles for the sound side, while the second row presents the results for the prosthetic side. The three columns, each consisting of two subplots, correspond to the three inclination-velocity combinations tested. In each subplot the results for both the Genium X3+Taleo (X3T) and the Genium X3+Evanto (X3E) are shown. The cycle duration was normalized to 100.

The flexion-extension ROM of the sound knee was higher compared to the prosthetic side in all the trials (Table 3). In addition, Table 3 shows an increasing trend of sound hip ROM with the Evanto foot across the three inclinations, with a statistically significant difference at the maximum inclination. In Figure 5 and Figure 6 the mean flexion-extension angles and the corresponding standard deviation bands for the hips and the knee, respectively, are summarized. 

### 3.3. Prosthesis Kinetics and Kinematics 

Positive knee torques result in knee extension, whereas negative knee torques result in knee flexion (Table 4). A positive ankle torque value indicates dorsi-extension, while a negative value is associated with dorsi-flexion. Positive axial load indicates weight-bearing, whereas negative axial load indicates unloading. Shank angular velocity positive values indicate knee flexion, while negative values correspond to knee extension (Table 5). The knee flexion-extension velocity’s positive value indicates a flexion movement, while negative values indicate an extension of the knee (Table 5). 

The prosthetic knee ROM (Table 3) evaluated with the external IMU setup, aligned closely with those recorded by the knee prosthesis’s embedded sensor (Table 5). Specifically, for the X3T combination, the external sensors overestimated the ROM by one degree compared to the embedded sensor for all the three inclinations (Table 3 and Table 5). Conversely, for the X3E combination, the external system underestimated the ROM by one degree compared to the embedded sensor at the first two inclinations (Table 3 and Table 5). This minimal difference indicates that the IMU sensor system and the methodology adopted provided a reliable estimation of the knee angle compared to the embedded sensors. Moreover, an offset between the two signals was observed in all six trials analyzed, probably due to the different processing conducted on the data. Specifically, in the Genium X3 prosthesis, a null value of flexion-extension angle corresponds to maximum extension without load. Thus, a full extension of the prosthesis when loaded resulted in negative flexion-extension angle values, which were not observed in the data from the external IMUs. The knee torque, ankle torque, and axial load were consistently higher for the X3E combination than for X3T combination, see maximum, minimum, and range values in Table 4. 

Statistically significant differences were found in the maximum knee torque, maximum axial load and maximum ankle torque all three inclinations (Table 4). Significant differences were also observed in the minimum axial load at 22%, and in the axial load range and ankle torque range at 8% and 17% (Table 4). Moreover, the knee torque range showed significant differences at all three inclinations (Table 4). Specifically, the X3E combination resulted in higher knee extension moments compared to the Taleo foot across all three inclinations. For both the X3T and X3E combinations, the maximum flexion-extension velocity was observed at an 8% incline. The positive peak of the shank angular velocity was significantly greater for the X3E at the first two inclinations (Table 5), while it was lower at the 22% inclination. However, this latter difference was not statistically significant (Table 5). Moreover, a significantly greater maximum shank angle was obtained with the use of the Evanto foot at the lowest inclination (Table 5).

## 4. Discussion

The aim of this exploratory case study was to analyze the biomechanics of an elite para standing athlete who performed diagonal roller skiing using two different prosthetic foot devices (Ottobock Taleo and Evanto), in combination with the Ottobock prosthetic knee Genium X3. This investigation on diagonal stride spatiotemporal parameters, hip and knee angles, and prosthesis kinetics and kinematics, revealed that the Evanto foot required greater active control from the user compared to the Taleo foot, as evidenced by increased knee and ankle torques and a more extended knee joint. However, the Evanto increased the foot swing phase across all three inclines and achieved higher peak angular velocity, which could potentially provide greater propulsion during the swing phase and enhance performance, particularly with adequate familiarization time.

Three different combinations of inclinations and speeds were tested (8% at 10 km/h, 17% at 7 km/h, and 22% at 6 km/h). Although the incline varied substantially between the last two conditions, the speeds selected based on the participant’s preference differed by only 1 km/h. This likely reflects the experienced participant’s pacing strategy rather than a direct scaling of velocity with incline. Increasing the sample size would enable the assessment of inter−subject variability and allow a more robust comparison of the two prosthetic configurations in the context of XC skiing. Additionally, the significant lack of studies regarding standing para skiers makes it challenging to compare the findings with existing literature. While results obtained for able-bodied athletes may not be fully applicable to paralympic athletes, they nevertheless could provide a useful reference point and serve as a basis for future investigations in this population. 

### 4.1. Spatiotemporal Parameters 

Elite able-bodied athletes tend to increase the percentage of the poling cycle spent in the swing phase to enhance performance, as suggested in [9]. Although, to the best of the authors’ knowledge, there is no confirmation that this is also valid for para-athletes, the use of the Evanto foot may be beneficial for performance as it consistently increased the foot swing phase across all three inclines compared to the Taleo foot (Table 2). Additionally, in [9] an increase in arm poling time was positively correlated to performance of able-bodied athletes. The participant exhibited a significantly longer arm poling phase when using the X3T combination, but only at the intermediate incline (Table 2). The observed differences in both foot swing and arm poling phase durations may be related to the enhanced mobility provided by the Evanto foot, which may facilitate limb swing and improve limb positioning for the subsequent contact phase, thus influencing overall movement coordination. Whether this translates into enhanced performance, as observed in able-bodied skiers [9], would require further investigation with direct measurements. Moreover, across both prosthetic combinations, cycle length decreased with increasing inclinations, consistent with previous research on able-bodied athletes [10]. 

### 4.2. Joint Angles

The hip flexion-extension ROM increased with the use of the X3E combination (Table 3), which could be beneficial for performance, as it may facilitate a more powerful push during the kick phase of the diagonal stride, as literature on able athletes suggests [9]. At 8% and 22%, the mean maximum knee flexion angle achieved with the X3E combination was significantly lower than the one obtained with the Taleo foot (Table 3). Indeed, the overall knee and hip joints behavior is more extended throughout the movement than with the Taleo foot at these two inclinations. This is reflected by the observed lower mean flexion angles: the mean hip flexion-extension curve for the X3E was consistently lower than that of the X3T configuration, except at the 17% inclination (Figure 5 and Figure 6). This adjustment might be due to the athlete’s need for greater effort to stabilize the foot, as the Evanto foot might have felt more unstable compared to the Taleo foot. Specifically, the athlete was not accustomed to skiing with it and the joint might have been extended more to provide a more stable base and to prevent the prosthesis from buckling.

### 4.3. Prosthetic Kinetics and Kinematics

An increased knee torque when using the X3E combination was found in this study (Table 4). This may be due to the athlete’s lack of training with the Evanto foot, leading the participant to exert greater force to enhance stability and control. In addition, the simultaneous increase in ankle torque suggests that additional load was placed on the prosthetic ankle joint to stabilize the movement. Therefore, the Evanto foot, compared to the Taleo, appears to require greater active control from the user, who may perceive compromised stability when switching to a multi-axial foot. The athlete’s subjective perception aligned with these findings: while reporting a more powerful push-off with the X3E, he also noted increased difficulty in controlling knee and hip dynamics, particularly at the steepest inclination where he expressed less satisfaction with X3E compared to X3T. Moreover, the increase in the axial load, when using the Evanto foot, indicates an overall higher force applied on the prosthetic side and greater involvement of both joints. Although a greater involvement of the prosthetic side may be beneficial in reducing the load on the contralateral limb, the dynamic of the sound limb was not analysed and thus this hypothesis cannot be verified. Further investigation into hip load and sound limb load is needed to fully understand the compensatory strategies used by this specific study participant. Finally, the higher peak angular velocity obtained with the use of the Evanto foot, may provide greater propulsion during the swing phase. 

Independently of the prosthetic configuration, axial load values increased at increasing inclinations (Table 4), possible because higher forces applied on the feet during the steepest inclines may be necessary to obtain the same grip [42]. A similar trend was observed for both knee and ankle torque (Table 4), suggesting a systematic increase in joint loading demands across the kinetic chain as slope steepness increases.

### 4.4. Study Limitations

One limitation of this study is the single-subject design. Consequently, our findings cannot be generalized to the broader population of standing para cross-country skiers or to other prosthetic configurations. The observed biomechanical patterns and compensation strategies are specific to this individual athlete and may be influenced by factors such as residual limb characteristics, level of amputation, training experience, and the mechanical properties of the prostheses used. 

An additional limitation of this study was the inability to fully correct drift errors in the calculation of the foot orientation, which consequently affected the calculation of ankle angles. This may be due to several causes, such as the low speeds used [34], noise introduced by the treadmill or incorrect positioning of the inertial sensors [43]. In particular, the continuous treadmill motion at the beginning of each trial may have hindered the drift correction of the feet orientation in the employed method. Nevertheless, this limitation did not affect the knee and hip angles. This might be because the sensors positioned on the feet were excluded from the assessment of the local reference frame procedure [35]. Furthermore, since the foot segment is in direct contact with the treadmill, it is more exposed to low-frequency oscillations at the beginning of each acquisition. As a result, the impact of low-frequency noise was likely more significant on these sensors compared to those placed on the shanks and thighs during the integration procedure. Further refinement and optimization of the procedure to account for dynamic conditions could therefore improve the accuracy of this evaluation.

Additionally, the limited training period with the Evanto prosthetic foot may have influenced the observed load distribution and control strategies. A longer period of familiarization with the Evanto prosthetic foot could also improve the comparability between the accustomed prosthesis and the newly introduced prosthetic foot.

## 5. Conclusions and Future Perspectives

To the best of the author’s knowledge, this study was the first with the aim to conduct a comprehensive analysis of the movement of a para standing XC skier. The devices tested included a knee prosthesis (Ottobock Genium X3) and two different foot prostheses (Ottobock Taleo and Ottobock Evanto). This study shows that the Genium X3+Evanto combination results in increased time spent in the foot swing phase and greater mobility of the lower limb joints compared to the Genium X3+Taleo when used by an athlete across various inclinations and speeds during XC skiing. These biomechanical changes, particularly the extended swing phase duration and higher joint range of motion, could imply potential benefits in the performance of XC diagonal skiing. However, these findings should be evaluated further by including physiologic measures and injury-related parameters to confirm any efficiency gains. The greater joint mobility observed with the multi-axial foot also results in higher joint load during skiing. With further practice and familiarization with the foot, this athlete may feel more confident, which could allow for increased push and greater ROM angles. Moreover, the proposed experimental setup and methodology may be utilized to further study XC skiing and to compare different prosthetic devices. 

Future studies should focus on conducting comprehensive biomechanical analyses of para XC standing skiers, including larger sample sizes to enable the evaluation of inter-subject variability and allow robust analyses of prosthetic performance in standing XC skiing. Parameters influencing performance in this para sport should be further investigated to better understand their role in efficiency and technique. Additionally, the inclusion of sound limb kinetics analysis would provide a more complete understanding of the global biomechanical adaptations and compensatory strategies adopted by prosthetic users during XC skiing. Finally, comparative studies between able-bodied and Paralympic athletes could provide more targeted insights for performance enhancement in standing para XC skiing and prosthetic design.

## Figures and Tables

**Table 1:** Placement of IMUs on body segments, sensor reference numbers, and data collected in the selected acquisition mode. Acc = Acceleration; Ang Vel = Angular Velocity.

Body Segment	Sensor No.	Mode
Sternum	1	IMU Acc + Ang Vel
Right Wrist	2	IMU Acc + Ang Vel
Left Thigh	3	IMU Acc + Ang Vel
	4	Orientation
Left Shank	5	IMU Acc + Ang Vel
	6	Orientation
Left Foot	7	IMU Acc + Ang Vel
	8	Orientation
Prosthetic Foot	9	IMU Acc + Ang Vel
	10	Orientation
Prosthesis Shaft	11	IMU Acc + Ang Vel
	12	Orientation
Socket	13	IMU Acc + Ang Vel
	14	Orientation
Sacrum	15	IMU Acc + Ang Vel
	16	Orientation

**Table 2:** Mean and standard deviation (Std) of cycle temporal parameters for the prosthetic limb and for the right arm calculated across cycles within each trial. Shown are percentages of the total cycle (%TC) and absolute time intervals. Statistical significance between X3T and X3E is indicated trough asterisks: * *p*-value < 0.05, ** *p*-value < 0.01, *** *p*-value < 0.001.

		Mean ± Std		
		10 km/h, 8% incline	7 km/h, 17% incline	6 km/h, 22% incline
Foot Contact Phase (%TC)	X3T	66 ± 8 *	66 ± 5 **	74 ± 5 ***
	X3E	59 ± 3	59 ± 3	62 ± 4
Foot Swing Phase (%TC)	X3T	34 ± 8 *	34 ± 5 **	26 ± 5 ***
	X3E	41 ± 3	41 ± 3	38 ± 4
Arm Poling Phase (%TC)	X3T	55 ± 4	52 ± 2 *	55 ± 3
	X3E	56 ± 2	50 ± 2	44 ± 3
Arm Recovery Phase (%TC)	X3T	45 ± 4	48 ± 2	45 ± 3
	X3E	44 ± 2	50 ± 2	56 ± 3
Foot Contact Phase (s)	X3T	0.76 ± 0.08	0.80 ± 0.05	0.90 ± 0.06 ***
	X3E	0.74 ± 0.07	0.74 ± 0.06	0.78 ± 0.06
Foot Swing Phase (s)	X3T	0.40 ± 0.12	0.42 ± 0.05 **	0.31 ± 0.07 ***
	X3E	0.51 ± 0.03	0.51 ± 0.03	0.47 ± 0.05
Arm Poling Phase (s)	X3T	0.52 ± 0.05	0.58 ± 0.04	0.66 ± 0.03
	X3E	0.54 ± 0.05	0.62 ± 0.03	0.70 ± 0.04
Arm Recovery Phase (s)	X3T	0.64 ± 0.07	0.64 ± 0.04 **	0.55 ± 0.04
	X3E	0.71 ± 0.03	0.62 ± 0.03	0.55 ± 0.05

**Table 3:** Mean and standard deviation (Std) of maximum, minimum and ROM values for hip and knee flexion-extension angles (deg) calculated across cycles within each trial. Statistical significance between the Genium X3+Taleo (X3T) and the Genium X3+Evanto (X3E) is indicated through asterisks: * *p*-value < 0.05, ** *p*-value < 0.01, *** *p*-value < 0.001.

		Mean ± Std								
		10 km/h, 8% incline			7 km/h, 17% incline			6 km/h, 22% incline		
		Max	Min	ROM	Max	Min	ROM	Max	Min	ROM
Sound Hip (deg)	X3T	38 ± 3 **	−24 ± 3 ***	62 ± 4	38 ± 3 ***	−25 ± 3 ***	65 ± 5	51 ± 4 *	−15 ± 2 ***	66 ± 4 *
	X3E	39 ± 3	−29 ± 3	67 ± 4	54 ± 4	−14 ± 2	67 ± 4	50 ± 4	−16 ± 2	67 ± 6
Sound Knee (deg)	X3T	41 ± 3 ***	8 ± 3 ***	33 ± 5 *	42 ± 2 ***	5 ± 2 ***	38 ± 2	38 ± 1	2 ± 1 *	36 ± 1
	X3E	37 ± 3	4 ± 2	33 ± 3	39 ± 1	4 ± 2	35 ± 3	41 ± 2	5 ± 2	36 ± 2
Prosthetic Hip (deg)	X3T	44 ± 3 ***	−16 ± 4 ***	60 ± 3 *	44 ± 3 ***	−16 ± 4 ***	66 ± 2 *	64 ± 6 ***	−3 ± 4 ***	67 ± 4 *
	X3E	39 ± 3	−23 ± 2	62 ± 4	42 ± 1	−23 ± 2	65 ± 3	41 ± 3	−26 ± 2	68 ± 2
Prosthetic Knee (deg)	X3T	27 ± 1 ***	1 ± 1 ***	25 ± 1	25 ± 1 *	1 ± 1	24 ± 1 *	25 ± 1 **	1 ± 1	25 ± 1
	X3E	23 ± 1	1 ± 1	23 ± 1	24 ± 2	1 ± 1	23 ± 1	28 ± 1	4 ± 1	24 ± 1

**Table 4:** Mean values and standard deviation (Std) of knee torque, axial load, and ankle torque maximum (Max), minimum (Min), and range (Δ), calculated across cycles within each trial, for Genium X3+Taleo (X3T) and Genium X3+Evanto (X3E). Statistical significance between X3T and X3E is indicated through asterisks: * *p*-value < 0.05, ** *p*-value < 0.01, *** *p*-value < 0.001.

		Mean ± Std								
		10 km/h, 8% incline			7 km/h, 17% incline			6 km/h, 22% incline		
		Max	Min	Δ	Max	Min	Δ	Max	Min	Δ
Knee Torque (N∙m)	X3T	87 ± 27 **	−24 ± 6	112 ± 30 **	128 ± 10 **	−17 ± 3	145 ± 13 **	148 ± 14 *	−16 ± 2	164 ± 15 *
	X3E	113 ± 13	−24 ± 4	150 ± 14	168 ± 14	−18 ± 3	168 ± 14	160 ± 16	−15 ± 2	175 ± 16
Axial Load (N)	X3T	744 ± 6 **	−134 ± 15	878 ± 58 *	863 ± 63 **	−93 ± 23	957 ± 61 **	951 ± 107 *	−97 ± 6 **	1048 ± 107
	X3E	822 ± 65	−125 ± 11	947 ± 71	996 ± 107	−97 ± 6	1093 ± 109	1027 ± 89	−85 ± 5	1112 ± 88
Ankle Torque (N∙m)	X3T	77 ± 18 **	−33 ± 7	110 ± 21 *	110 ± 8 **	−25 ± 5	136 ± 13 *	119 ± 10 *	−25 ± 3 **	144 ± 11
	X3E	104 ± 12	−30 ± 6	134 ± 15	137 ± 11	−24 ± 3	161 ± 11	140 ± 9	−25 ± 4	165 ± 11

**Table 5:** Mean and standard deviation (Std) of shank angular velocity, knee flexion-extension velocity, shank angle, and knee angle maximum (Max), minimum (Min), and range (Δ), calculated across cycles within each trial, for Genium X3+Taleo (X3T) and Genium X3+Evanto (X3E). Statistical significance between X3T and X3E is indicated through asterisks: * *p*-value < 0.05, ** *p*-value < 0.01, *** *p*-value < 0.001.

		Mean ± Std								
		10 km/h, 8% incline			7 km/h, 17% incline			6 km/h, 22% incline		
		Max	Min	Δ	Max	Min	Δ	Max	Min	Δ
Shank Angular Velocity (deg/s)	X3T	241 ± 47 **	−223 ± 70	464 ± 103	207 ± 13 *	−232 ± 20	439 ± 18	187 ± 12 *	−240 ± 27	427 ± 9
	X3E	265 ± 9	−233 ± 21	497 ± 24	217 ± 12	−232 ± 20	439 ± 21	182 ± 9	−234 ± 31	416 ± 29
Knee flexion−extension velocity (deg/s)	X3T	225 ± 40	−165 ± 45	389 ± 64	185 ± 24 *	−178 ± 43	363 ± 61	186 ± 19 *	−186 ± 37	372 ± 28
	X3E	214 ± 17	−148 ± 41	361 ± 42	177 ± 22	−183 ± 41	360 ± 42	171 ± 12	−194 ± 20	364 ± 20
Shank Angle (deg)	X3T	57 ± 15 **	−11 ± 2	69 ± 16 *	50 ± 2	−16 ± 2	66 ± 3	45 ± 2 *	−18 ± 2	63 ± 2
	X3E	65 ± 3	−12 ± 3	77 ± 3	50 ± 1	−15 ± 1	66 ± 1	42 ± 2	−19 ± 2	61 ± 2
Knee Angle (deg)	X3T	22 ± 1	−2 ± 1 **	24 ± 1	21 ± 1	−3 ± 0.1 **	23 ± 1	21 ± 0.42	−3 ± 0.2	24 ± 1
	X3E	22 ± 1	−2 ± 0.2	24 ± 1	21 ± 1	−3 ± 0.2	24 ± 1	21 ± 1	−3 ± 0.1	24 ± 1

## Data Availability

The data presented in this study are available on request from the corresponding author.

## References

[1] Vanlandewijck Y.C., Thompson W.R. (2011). The Paralympic Athlete—Handbook of Sports Medicine and Science.

[2] Gastaldi L. Research in Paralympic XC-Skiing. Proceedings of the ICSNS 2015 3rd International Congress on Science and Nordic Skiing.

[3] International Paralympic Committee (2016). Explanatory Guide to Paralympic Classifcation—Paralympic Winter Sports.

[4] Carlsen C.H., Baumgart J.K., Kocbach J., Haugnes P., Paulussen E.M.B., Sandbakk Ø. (2021). Framework for In-Field Analyses of Performance and Sub-Technique Selection in Standing Para Cross-Country Skiers. Sensors.

[5] Solli G.S., Kocbach J., Seeberg T.M., Tjønnås J., Rindal O.M.H., Haugnes P., Torvik P.Ø., Sandbakk Ø. (2018). Sex-Based Differences in Speed, Sub-Technique Selection, and Kinematic Patterns during Low- and High-Intensity Training for Classical Cross-Country Skiing. PLoS ONE.

[6] Sandbakk Ø., Losnegard T., Skattebo Ø., Hegge A.M., Tønnessen E., Kocbach J. (2016). Analysis of Classical Time-Trial Performance and Technique-Specific Physiological Determinants in Elite Female Cross-Country Skiers. Front. Physiol..

[7] Ettema G., Kveli E., Øksnes M., Sandbakk Ø. (2017). The Role of Speed and Incline in the Spontaneous Choice of Technique in Classical Roller-Skiing. Hum. Mov. Sci..

[8] Nilsson J., Tveit P., Eikrehagen O., Nilsson J. (2004). Cross-Country Skiing: Effects of Speed on Temporal Patterns in Classical Style and Freestyle Cross-country Skiing. Sports Biomech..

[9] Lindinger S.J., Göpfert C., Stöggl T., Müller E., Holmberg H.C. (2009). Biomechanical Pole and Leg Characteristics during Uphill Diagonal Roller Skiing. Sports Biomech..

[10] Bernard B., Boulay Marcel R., Benoit R. (1992). Propulsive and Gliding Phases in Four Cross-Country Skiing Techniques. Med. Sci. Sports Exerc..

[11] De Luigi A.J., Cooper R.A. (2014). Adaptive Sports Technology and Biomechanics: Prosthetics. PM&R.

[12] Hadj-Moussa F., Ngan C.C., Andrysek J. (2022). Biomechanical Factors Affecting Individuals with Lower Limb Amputations Running Using Running-Specific Prostheses: A Systematic Review. Gait Posture.

[13] Rice I., Hettinga F.J., Laferrier J., Sporner M.L., Heiner C.M., Burkett B., Cooper R.A. (2010). Biomechanics. The Paralympic Athlete: Handbook of Sports Medicine and Science.

[14] Toumi A., Simoneau-Buessinger É., Bassement J., Barbier F., Gillet C., Allard P., Leteneur S. (2021). Standing Posture and Balance Modalities in Unilateral Transfemoral and Transtibial Amputees. J. Bodyw. Mov. Ther..

[15] Fuchs K., Krauskopf T., Lauck T.B., Klein L., Mueller M., Herget G.W., Von Tscharner V., Stutzig N., Stieglitz T., Pasluosta C. (2021). Influence of Augmented Visual Feedback on Balance Control in Unilateral Transfemoral Amputees. Front. Neurosci..

[16] Claret C.R., Herget G.W., Kouba L., Wiest D., Adler J., Von Tscharner V., Stieglitz T., Pasluosta C. (2019). Neuromuscular Adaptations and Sensorimotor Integration Following a Unilateral Transfemoral Amputation. J. Neuroeng. Rehabil..

[17] Kannenberg A., Zacharias B., Pröbsting E. (2014). Benefits of Microprocessor-Controlled Prosthetic Knees to Limited Community Ambulators: Systematic Review. J. Rehabil. Res. Dev..

[18] Berry D., Olson M.D., Larntz K. (2009). Perceived Stability, Function, and Satisfaction among Transfemoral Amputees Using Microprocessor and Nonmicroprocessor Controlled Prosthetic Knees: A Multicenter Survey. J. Prosthet. Orthot..

[19] Rogers-Bradley E. (2023). Design and Evaluation of a Quasi-Passive Variable Stiffness Ankle-Foot Prosthesis to Improve Biomechanics Across Walking Speeds. Ph.D. Thesis.

[20] Paradisi F., Delussu A.S., Brunelli S., Iosa M., Pellegrini R., Zenardi D., Traballesi M. (2015). The Conventional Non-Articulated SACH or a Multiaxial Prosthetic Foot for Hypomobile Transtibial Amputees? A Clinical Comparison on Mobility, Balance, and Quality of Life. Sci. World J..

[21] Baumgart J.K., Kocbach J., Podolski M., Sandbakk Ø., Severin A.C. (2025). Effect of Class, Sex, and Final Rank on the Time Distribution Across Terrains During Para Cross-Country Skiing Races. Eur. J. Sport. Sci..

[22] Carlsen C.H., Severin C., Sandbakk Ø., Baumgart J.K. (2022). Comparison of Race Time-Differences Between and Within Para and Able-Bodied Cross-Country Skiers. Front. Sports Act. Living.

[23] Holmberg L.J., Ohlsson M.L., Danvind J. (2012). Musculoskeletal Simulations: A Complementary Tool for Classification of Athletes with Physical Impairments. Prosthet. Orthot. Int..

[24] Bragaru M., Dekker R., Geertzen J.H.B., Dijkstra P.U. (2011). Amputees and Sports A Systematic Review. Sports Med..

[25] Poonsiri J., Dekker R., Dijkstra P.U., Hijmans J.M., Geertzen J.H.B. (2018). Bicycling Participation in People with a Lower Limb Amputation: A Scoping Review. BMC Musculoskelet. Disord..

[26] Demšar I., Duhovnik J., Lešnik B., Supej M. (2015). Multi-Axis Prosthetic Knee Resembles Alpine Skiing Movements of an Intact Leg. J. Sports Sci. Med..

[27] Otto Bock Healthcare Gmbh Genium X3 3B5-2/3B5-2=ST—Instruction for Use (Qualified Personnel).

[28] Mileusnic M.P., Rettinger L., Highsmith M.J., Hahn A. (2021). Benefits of the Genium Microprocessor Controlled Prosthetic Knee on Ambulation, Mobility, Activities of Daily Living and Quality of Life: A Systematic Literature Review. Disabil. Rehabil. Assist. Technol..

[29] Otto bock Healthcare Gmbh Taleo 1C50/1C53 LP—Instruction for Use (Qualified Personnel).

[30] Otto Bock Healthcare Gmbh Evanto—Instruction for Use (Qualified Personnel).

[31] Maciejasz P., Budny T., Sauer M., Umari M., Korber J., Ernst J., Altenburg B., Hahn A., Braatz F. (2024). User Preference and Patient Benefits of a Novel Energy Storing and Return Foot: A Randomized, Cross-over Clinical Trial. Prosthet. Orthot. Int..

[32] Delsys (2021). Trigno^®^ Wireless Biofeedback System User’s Guide 2021.

[33] Manz S., Seifert D., Altenburg B., Schmalz T., Dosen S., Gonzalez-Vargas J. (2023). Using Embedded Prosthesis Sensors for Clinical Gait Analyses in People with lower Limb Amputation: A Feasibility Study. Clin. Biomech..

[34] Fasel B., Spörri J., Schütz P., Lorenzetti S., Aminian K. (2017). Validation of Functional Calibration and Strap-down Joint Drift Correction for Computing 3D Joint Angles of Knee, Hip, and Trunk in Alpine Skiing. PLoS ONE.

[35] Caruso M., Digo E., Gastaldi L., Pastorelli S., Cereatti A. (2024). A Constrained-Based Optimization Method for Real-Time Kinematics Using Magneto-Inertial Signals: Application to Upper Limb Joint Angles Estimation during Prolonged Recordings. IEEE Access.

[36] Fasel B., Favre J., Chardonnens J., Gremion G., Aminian K. (2015). An Inertial Sensor-Based System for Spatio-Temporal Analysis in Classic Cross-Country Skiing Diagonal Technique. J. Biomech..

[37] Sabatini A.M. (2005). Quaternion-Based Strap-down Integration Method for Applications of Inertial Sensing to Gait Analysis. Med. Biol. Eng. Comput..

[38] Favre J., Jolles B.M., Siegrist O., Aminian K. (2006). Quaternion-Based Fusion of Gyroscopes and Accelerometers to Improve 3D Angle Measurement. Electron. Lett..

[39] Digo E., Pierro G., Pastorelli S., Gastaldi L. (2020). Evaluation of Spinal Posture during Gait with Inertial Measurement Units. Proc. Inst. Mech. Eng. H.

[40] Siciliano B., Sciavicco L., Villani L., Oriolo G. (2009). Robotics: Modelling, Planning and Control.

[41] Benjamini Y., Hochberg Y. (1995). Controlling the False Discovery Rate: A Practical and Powerful Approach to Multiple Testing. J. R. Stat. Soc. Ser. B (Methodol.).

[42] Andersson E. (2016). Physiological and Biomechanical Factors Determining Cross-Country Skiing Performance. Ph.D. Thesis.

[43] Iosa M., Picerno P., Paolucci S., Morone G. (2016). Wearable Inertial Sensors for Human Movement Analysis. Expert Rev. Med. Devices.

